# Selective Feticide in a Dichorionic Twin Pregnancy After IVF Following Diagnosis of Trisomy 21 in One Twin: A Case Report

**DOI:** 10.7759/cureus.99718

**Published:** 2025-12-20

**Authors:** Bedrana Muracevic-Begovic, Amra Mujkanovic-Dzino, Jasmin Hodzic

**Affiliations:** 1 Obstetrics and Gynecology, Cantonal Hospital Zenica, Zenica, BIH; 2 Obstetrics and Gynecology, General Hospital Konjic, Konjic, BIH; 3 Obstetrics and Gynecology, Polyclinic Medicom, Zenica, BIH

**Keywords:** ivf, prenatal diagnosis, selective feticide, trisomy 21, twin pregnancy

## Abstract

In multiple pregnancies complicated by a severe fetal anomaly, selective feticide may be performed to reduce maternal risks and improve the prognosis of the unaffected twin. We present the case of a 38-year-old patient who conceived through in vitro fertilization (IVF) and was diagnosed with a dichorionic diamniotic twin pregnancy discordant for trisomy 21. Non-invasive prenatal testing (NIPT) indicated a high risk for trisomy 21 in the second twin, which was subsequently confirmed by amniocentesis and molecular genetic testing. The first twin demonstrated a normal male karyotype, whereas the second twin exhibited a male karyotype with trisomy 21. Given that selective feticide is not legally allowed in Bosnia and Herzegovina, the patient was referred to the Clinical Center of Serbia in Belgrade. Following ethical approval, selective feticide of the affected twin was performed at 33+1 weeks of gestation. The cesarean delivery at 33+6 weeks resulted in one live male neonate (2,440 g, Apgar score 6) and one stillborn male (1,900 g, Apgar score 0). The maternal postoperative course was uneventful, and the surviving infant demonstrated normal growth and neurodevelopment at one-month follow-up. This case underscores the importance of early genetic screening, multidisciplinary counseling, and awareness of legal and ethical frameworks that may necessitate cross-border management in twin pregnancies discordant for chromosomal abnormalities.

## Introduction

Twin pregnancies, especially those conceived through in vitro fertilization (IVF), carry a higher risk of perinatal complications compared with singleton pregnancies, including preterm birth, growth discordance, and chromosomal abnormalities [1]. Advances in prenatal screening, such as non-invasive prenatal testing (NIPT) and detailed ultrasound evaluation, have greatly improved early detection of fetal aneuploidy, even in multiple gestations [2].

When a severe chromosomal abnormality, such as trisomy 21, is identified in one fetus of a dichorionic twin pregnancy, selective fetal feticide may be offered to optimize outcomes for the unaffected co-twin and reduce maternal risk [3]. This procedure is generally associated with a favorable prognosis for the surviving fetus, particularly in dichorionic twins, although it raises complex ethical and psychological considerations [4].

Clinical management requires careful assessment of gestational age, chorionicity, and maternal health, alongside adherence to local legal frameworks and institutional ethics guidelines [5]. Selective fetal feticide is typically performed via ultrasound-guided intracardiac injection of potassium chloride (KCl), with timing and technique tailored to minimize risk to the remaining twin [6].

With the increasing use of assisted reproductive technologies and the resulting rise in multiple pregnancies, understanding the indications, techniques, and outcomes of selective fetal feticide is essential for contemporary obstetric practice [2,3]. This case report describes the clinical course, diagnostic evaluation, and management of a dichorionic twin pregnancy complicated by trisomy 21 in one fetus, culminating in selective feticide and delivery.

## Case presentation

A 38-year-old patient presented with a dichorionic diamniotic twin pregnancy conceived via in vitro fertilization (IVF). Her obstetric history included one prior IVF-conceived twin pregnancy that resulted in the delivery of healthy twins by cesarean section at 36 weeks of gestation, nine years earlier. She had no previous miscarriages and no significant family history. It was noted, however, that her sister-in-law’s child has Down syndrome. At 12+4 weeks of gestation, routine fetal screening revealed that twin II had an absent nasal bone and a nuchal translucency of 3 mm [7], while all measurements for twin I were within normal limits.

At 13+1 weeks, non-invasive prenatal testing (NIPT) [2] indicated a high risk for trisomy 21 in twin II. Amniocentesis at 16+3 weeks confirmed trisomy 21 (male) in twin II and a normal diploid karyotype (male) in twin I. A confirmatory molecular genetic analysis (QF-PCR and chromosomal microarray, CMA) [8,9] of the amniotic fluid performed in Belgrade at 20+6 weeks again verified trisomy 21 in twin II.

**Figure 1:**
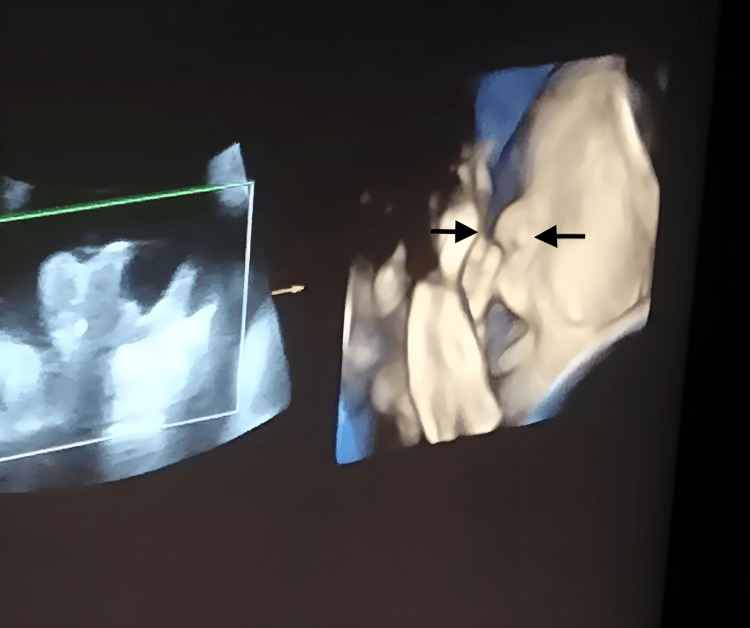
4D Ultrasound view of twin II at 31 weeks of gestation demonstrating absence of the nasal bone. The 4D ultrasound shows the facial anatomy of twin II at 31 weeks of gestation. The nasal bone is not visualized in the expected region, consistent with its absence [7].

Subsequent serial ultrasound evaluations revealed reduced fetal movements, oligohydramnios, and an absent nasal bone in twin II. Figure 1 demonstrates the twin II at 31 weeks of gestation with an absent nasal bone. All the parameters for twin I remained within normal limits.

Because selective feticide was forbidden by national legal regulations in Bosnia and Herzegovina, the patient requested referral to a tertiary center in Belgrade for further management. The Ethics Committee of the Clinical Center of Serbia reviewed the case and approved selective feticide on maternal request for medical indications.

At 33+1 weeks of gestation, the patient underwent ultrasound-guided selective feticide of twin II with an injection of 8 mL of 7.45% KCl into the fetal heart, followed by sonographic confirmation of fetal asystole on the same day.

At 33+6 weeks, due to regular uterine contractions and obstetric indications, a cesarean section was performed. The procedure resulted in the delivery of a live male infant (twin I) weighing 2,440 g, measuring 44 cm, with an Apgar score of 6, and a stillborn male infant (twin II) weighing 1,900 g, measuring 45 cm, with an Apgar score of 0 [10].

The postoperative course was uneventful, and the patient was discharged on the 11th postoperative day. At one-month follow-up, the surviving infant demonstrated appropriate growth and development. Subsequent prenatal investigations, genetic testing, and interventions are summarized in Table 1.

Nuchal translucency (NT) measurement and nasal bone assessment were performed according to the Fetal Medicine Foundation (FMF) guidelines, which are freely accessible for clinical use and do not require licensing. The Apgar scoring system used for neonatal assessment is a standardized clinical tool that is freely available. Diagnostic methodologies, including NIPT, QF-PCR, and chromosomal microarray analysis (CMA), were applied using validated and widely accepted protocols. Appropriate methodological references for these tools and scoring systems have been added to the reference list.

**Table 1:** Chronological summary of prenatal findings, interventions, and outcomes in the current twin pregnancy NIPT: non-invasive prenatal testing [2]; NT: nuchal transluency measurement (FMF) [7]; Nasal bone assessment (FMF) [7]; QF-PCR: quantitative fluorescent polymerase chain reaction [8]; CMA: chromosomal microarray analysis [9]; AS: Apgar score [10]; KCl: potassium chloride

Gestational Age / Time	Event / Finding	Investigation / Intervention	Outcome / Note
12+4 weeks	Routine fetal screening	Ultrasound	Twin II: absent nasal bone [7], NT 3 mm [7]; Twin I: all measurements normal
13+1 weeks	Prenatal screening	NIPT [2]	Twin II: high risk for trisomy 21
16+3 weeks	Diagnostic testing	Amniocentesis	Twin II: trisomy 21 (male); Twin I: normal diploid karyotype (male)
20+6 weeks	Confirmatory genetic testing	QF-PCR + CMA (amniotic fluid) [8, 9]	Trisomy 21 confirmed in Twin II
20–33 weeks	Serial ultrasound monitoring	Ultrasounds	Twin II: reduced movements, oligohydramnios; Twin I: normal findings
33+1 weeks	Selective feticide	Ultrasound-guided injection of 8 mL 7.45% KCl into the heart of Twin II	Fetal asystole in Twin II was confirmed the same day
33+6 weeks	Delivery	Cesarean section	Twin I: live male, 2440 g, 44 cm, AS 6; Twin II: stillborn male, 1900 g, 45 cm [10]
Post-operative	Maternal follow-up	Hospitalization	Uncomplicated course; discharged on postoperative day 11
1 month	Infant follow-up	Clinical examination	Twin I shows appropriate growth and development

## Discussion

Selective feticide in dichorionic twin pregnancies requires careful consideration of medical, ethical, and legal aspects. Dichorionic twins allow safer selective feticide than monochorionic twins because each fetus has its own placenta, reducing the risk to the co-twin [11]. Early detection of chromosomal anomalies, such as trisomy 21, through NIPT and confirmatory amniocentesis is critical to allow timely intervention and minimize procedure-related complications [2,6].

Studies have shown that selective fetal feticide, when performed after appropriate counseling and ethical approval, improves outcomes for the unaffected twin, including higher survival rates and a reduced risk of preterm delivery [11,12]. Intracardiac injection of KCl remains the most widely used technique in dichorionic gestations [6].

Our case highlights several important points: the intervention was performed at 33+1 weeks, cross-border coordination was required due to restrictive local legislation, and formal ethics committee approval was obtained.

From a legal and ethical perspective, trisomy 21 (Down syndrome) is not considered a routine legal indication for pregnancy termination in Serbia, particularly in advanced gestation. However, Serbian legislation allows pregnancy termination beyond standard gestational limits following individual case evaluation and formal approval by an institutional ethics committee. In the present case, selective feticide was approved by the Ethics Committee of the Clinical Center of Serbia based on maternal request, confirmed fetal diagnosis, advanced gestational age, and comprehensive multidisciplinary assessment.

The procedure was successful, with no adverse maternal outcomes and survival of the unaffected twin. At one-month follow-up, the surviving twin demonstrated normal growth and developmental milestones, consistent with previous reports [4,13].

In Bosnia and Herzegovina, selective feticide is prohibited by national legislation, necessitating referral to Serbia, where the procedure may be approved on a case-by-case basis following formal ethical review. This underscores the importance of multidisciplinary counseling, international collaboration, and adherence to both medical standards and patient autonomy. The clinicians should remain aware of local regulations and relevant international guidelines when planning selective fetal feticide [6].

Because this is a single case with short postpartum follow-up, the findings cannot be generalized to broader populations or used to establish safety or long-term outcomes of late selective feticide. The description is limited to the clinical course observed in this individual pregnancy.

## Conclusions

This case illustrates the practical challenges of managing dichorionic twin pregnancies with discordant aneuploidy in settings with restrictive legislation. The timing and conduct of selective feticide were determined by legal, ethical, and logistical constraints rather than clinical preference. As a single case with limited follow-up, this report cannot conclude on safety, long-term outcomes, or comparative effectiveness; instead, it highlights the need for timely access to multidisciplinary counseling and clear regulatory pathways.
